# From Antagonism to Coevolutionary Regulation: Multidimensional Phage–Biofilm Interaction Networks and Translational Targeted Anti-Infection Strategies

**DOI:** 10.3390/microorganisms14102290

**Published:** 2026-10-08

**Authors:** Yuxin Wang, Linjia Ma, Yahao Yu, Qi An, Yifang Cui, Bingyan Xue, Li Yi, Xiaokang Li, Baobao Liu, Yang Wang

**Affiliations:** 1 College of Animal Science and Technology, Henan University of Science and Technology, Luoyang 471000, China; 2 Henan Provincial Engineering Research Center for Detection and Prevention and Control of Emerging Infectious Diseases in Livestock and Poultry, Luoyang 471003, China; 3 College of Life Science, Luoyang Normal University, Luoyang 471934, China

**Keywords:** phage, biofilm, phage–biofilm interaction, antagonism, synergism, phage therapy

## Abstract

Bacterial biofilms possess inherent physical barriers and structural heterogeneity. These features pose a major obstacle to clinical anti-infective therapy. This review systematically examines the dynamic interplay between phages and bacterial biofilms, which ranges from antagonistic competition to synergistic interaction. It aims to dissect the underlying mechanisms and translational potential of phages as next-generation precision anti-infective agents. A core scientific question in phage–biofilm interactions is how phages penetrate and remodel the three-dimensional extracellular matrix of biofilms. Classic mechanisms include direct lysis of bacteria embedded in biofilms, degradation of matrix scaffolds by phage-derived exopolysaccharide depolymerases, and modulation of bacterial virulence and biofilm phenotypes via lysogenic conversion. Auxiliary mechanisms encompass synergistic bactericidal effects of phages and the host’s innate immune system, as well as niche competition within microbial communities. Recently identified mechanisms involve phage-mediated interference with bacterial quorum sensing, physical remodeling of biofilm spatial architecture, and enhanced antibiotic penetration across biofilm barriers. These processes collectively shape the microenvironmental network linking phages, bacteria, and host cells. By synthesizing the complex phage–biofilm interplay, this review provides a theoretical basis for designing engineered phage cocktails, optimizing phage–antibiotic combination regimens, and developing intelligent biomaterial delivery systems. These delivery systems are intended for targeted treatment of chronic biofilm-associated infections.

## 1. Background

Biofilm formation is a primary driver of persistent bacterial infections and global antibiotic resistance in human and veterinary clinical settings. Therefore, developing biofilm-targeted therapeutics is a critical challenge in antibacterial control research [1,2]. Biofilms form a physical barrier through complex extracellular polymeric substances (EPSs). This EPS matrix shields bacteria from host immune clearance and markedly reduces antibiotic penetration and therapeutic efficacy. Consequently, biofilms cause intractable infectious disorders, such as cystic fibrosis-associated pulmonary infection, chronic wound infection, and implant-associated infection [3,4]. Conventional antibiotic monotherapy is increasingly failing, and drug-resistant pathogens frequently emerge. As a result, phage therapy has re-emerged as a promising alternative or adjuvant strategy for combating bacterial infections. Its advantages include precise host-specific lysis, self-replication, and the ability to access biofilm matrices. However, this penetration varies considerably among phages. It depends on matrix composition, bacterial physiology, and the presence of matrix-degrading depolymerases rather than being a uniform “innate” property [5,6]. For biofilm eradication, phages can directly infiltrate loosely structured biofilms at the early adhesion and late dispersion stages and lyse embedded bacteria. Moreover, many phages encode tail-spike-associated exopolysaccharide depolymerases. These enzymes disrupt EPS integrity and disassemble mature biofilm matrices [3,7]. Accumulating in vitro and in vivo evidence has shown that virulent phages that target critical pathogens, such as *Acinetobacter baumannii* and *Salmonella enteritidis*, exert robust anti-biofilm activity [8,9,10]. In addition, phage-derived endolysins, such as Lys572 against *Streptococcus suis*, show unique advantages in food preservation and local treatment because of their high stability and strong ability to eliminate mature biofilms [11]. Nevertheless, emerging evidence indicates that the interaction between phages and biofilms is not simply antagonistic. Instead, it is a complex dynamic interaction network ranging from antagonism to synergy [12,13].

Phages differ from conventional antibacterial agents. A defining feature is their capacity for reciprocal adaptive evolution with bacteria under survival stress. This coevolution involves phenotypic changes and genetic mutations. It persists even within biofilms [13]. In addition, phages extensively regulate bacterial social behaviors and remodel biofilm architecture through lysogenic conversion, by encoding quorum-sensing interfering molecules, or by producing regulatory proteins. Under specific conditions, they may even promote biofilm formation and alter its physicochemical properties [14]. This bidirectional regulation drives an ongoing arms-race-like coevolution. It profoundly influences the ecological and functional traits of microbial communities [12].

Existing studies have outlined multiple mechanisms by which phages regulate biofilms. These mechanisms include lysis, enzymatic degradation, quorum-sensing interference, and adaptive infection in response to bacterial metabolic states [4]. However, current knowledge remains fragmented. Systematic integration and in-depth discussion are lacking for several critical scientific issues. These issues include how phages precisely sense and respond to microenvironmental stresses within biofilms, such as hypoxia and nutrient limitation, how they interact synergistically or antagonistically with the host immune system to modulate biofilm status, and how to optimize phage–antibiotic combination strategies to eradicate biofilm-embedded bacteria [4,15,16]. This deficiency restricts comprehensive evaluation and rational development of phage therapy. Therefore, this study aimed to go beyond the traditional view of phages as simple biofilm eliminators. We systematically clarified their dynamic interactions with biofilms, ranging from antagonism to synergism. We focused on the core mechanisms by which phages break through the physical barrier of biofilms via matrix degradation and targeting dormant cells. We further dissected the molecular mechanisms underlying their regulation of biofilm formation, structure, and heterogeneity. We expect this work to provide a solid theoretical basis and a clear translational direction for developing engineered phages with broad host ranges and strong biofilm-penetrating capacity. We also expect it to guide the design of intelligent synergistic strategies integrating phages, antibiotics, and immunomodulators. It may guide the construction of efficient targeted delivery systems. These advances are needed to address the urgent clinical challenge of biofilm-associated infections [6,17,18].

Throughout this review, the terms “phage” and “phages” refer broadly to bacteriophages. The term “phage derivatives” denotes phage-encoded enzymes used independently of the intact virion. These enzymes are principally depolymerases, endolysins, and tail-associated enzymes. The terms “phage preparations” and “phage-based agents” include natural phages, phage cocktails, and engineered phages. “Phage therapy” refers to the experimental or clinical use of any of these agents against bacterial infection. We also use outcome terms with distinct meanings. The term “biofilm inhibition” refers to prevention of biofilm formation. The terms “biofilm disruption” and “biofilm removal” refer to physical breakup or detachment of an existing biofilm. The term “biomass reduction” refers to loss of biofilm mass. The term “bacterial killing” refers to reduction in viable counts. The term “biofilm eradication” refers to complete removal of viable cells and biofilm matrix. If a study reports only one of these outcomes, we do not generalize the finding to the others.

Literature search strategy: For this narrative review, we searched PubMed, Web of Science, and Scopus for English-language articles published up to 2026, using combinations of terms including “bacteriophage”, “phage therapy”, “biofilm”, “extracellular polymeric substance”, “phage–antibiotic synergy”, “endolysin”, “depolymerase”, “lysogenic conversion”, and “phage delivery”. Original research and key reviews were retained, with priority given to studies reporting quantitative biofilm outcomes; the selection is illustrative rather than systematic, and citations were chosen to illustrate mechanisms and translational evidence.

## 2. The Relationship Between Phage and Biofilm: Antagonism and Synergism

### 2.1. Biofilms Serve as a Physical Barrier for Bacteria Against Phage Infection

A dense matrix composed of EPS is central to bacterial biofilms. This matrix mainly consists of polysaccharides, proteins, lipids, and extracellular DNA (eDNA) [19], which together form a sophisticated three-dimensional network. To approach and adsorb to surface receptors of bacteria embedded in biofilms, phage particles must first penetrate this barrier. EPS components can markedly reduce phage diffusion through steric hindrance. They can even physically trap phage particles, preventing them from reaching target bacterial cells. Among these components, high-molecular-weight polysaccharides such as alginate form highly viscous gels. These gels greatly restrict the Brownian motion and convective transport of viral particles.

Beyond physical obstruction, EPS components may also exert nonspecific adsorption or repulsion effects on phages; this possibility warrants further exploration. Studies have revealed that EPS produced by *P. aeruginosa* strain OMCS-1 can interact with chromium, lead, and cadmium through electrostatic interactions and complexation mechanisms [20]. Phage capsid proteins generally carry negative charges [21]. Therefore, these charges may trigger electrostatic interactions with charged molecules such as eDNA within EPS. Such interactions can trap or repel phage particles. Such nonspecific binding or repulsion may deplete viable infectious phage particles and reduce phage infectivity. However, this hypothesis still requires further experimental verification.

As bacterial metabolism consumes oxygen and nutrients, hypoxic and nutrient-deficient regions frequently develop in the deeper layers of biofilms. This condition drives bacteria into a slow-metabolizing or dormant persister state. The unique microenvironment within biofilms further exacerbates the difficulty in phage infection [4]. Phage replication depends heavily on the active metabolism of host cells to supply energy and biosynthetic precursors. Such bacteria cease division and may downregulate the expression of surface receptors. Consequently, phages cannot adsorb efficiently. Even if they inject their genetic material, they may fail to initiate a productive replication cycle [4]. This physiological heterogeneity provides a refuge. It allows internal bacteria to evade complete elimination by phages. In addition, local pH, ionic strength, and metabolic by-products within biofilms may affect phage stability and activity. Certain phages are sensitive to pH fluctuations [22]. Microenvironmental acidification arising from bacterial fermentation inside biofilms may induce denaturation of phage capsid proteins or compromise structural integrity. Ultimately, this process can lead to phage inactivation.

Together, these physical, chemical, and physiological barriers make biofilms inherently resistant to phage therapy. This resistance represents one of the core obstacles that must be overcome in developing phage-based strategies for biofilm-associated infections. Elucidating the specific mechanisms underlying these antagonistic effects provides a basis for designing innovative therapeutic strategies. Such strategies should efficiently penetrate and dismantle biofilms.

### 2.2. Phages and Their Derivatives Can Effectively Lyse Biofilm

Because bacterial biofilms cause substantial harm, researchers have developed a range of physical, chemical, and biological approaches to eliminate them [23,24,25]. Current research priorities include the development of anti-adhesive surfaces [26], strategies to inhibit and reduce bacterial adhesion, antimicrobial photodynamic therapy [27], induction of biofilm detachment [28], interference with bacterial second messenger signaling mediated by cyclic di-AMP (c-di-AMP) and cyclic di-GMP (c-di-GMP) [29], degradation of outer membrane matrix components [30], and application of quorum sensing inhibitors [31]. Although these methods show some anti-biofilm activity, most remain limited in effectiveness. Phages are among the most actively investigated alternatives because they can disrupt biofilms, kill pathogenic bacteria, and undergo self-amplification. Both phages and their encoded enzymes have attracted attention as promising candidates for biofilm control and eradication [32]. Table 1 summarizes studies that assess the efficacy of phages and phage-derived products against bacterial biofilms. Across the studies summarized in Table 1, phage derivatives appear qualitatively more potent against preformed mature biofilms. We did not perform a formal meta-analysis or statistical comparison because the entries differ greatly in bacterial species, biofilm model, phage dose/multiplicity of infection (MOI), exposure time, and endpoints. Individual phages or phage cocktails can effectively clear established biofilms. However, biofilm-degrading enzymes, particularly depolymerases, appear to be the principal mediators of this effect. Some virulent phages do not naturally harbor depolymerases or other matrix-degrading enzymes. Combining these phages with other anti-biofilm agents is an effective strategy to disrupt mature biofilms.

### 2.3. Ecological and Evolutionary Significance of the Complex Interaction Network Between Phage and Biofilms

Although phages and phage-based preparations can effectively eliminate bacterial biofilms, it is necessary to consider their potential to promote biofilm formation. Accumulating studies have shown that phages act not only as biofilm destroyers but also as complex regulators of biofilm formation, structure, and dynamics. This dual role is driven largely by an ongoing arms race and coevolution [12,13]. Such evolutionary games are prevalent in environmental and clinical biofilms. For example, metagenomic analysis of biofilms from meat-processing-plant drainage systems has revealed the coexistence of phages targeting *Pseudomonas* and *Klebsiella* with host bacterial defense genes. In addition, the detection of phage-encoded anti-CRISPR proteins clearly illustrates the coevolutionary cycle of attack, defense, and counterattack [13]. High mutation rates and phenotypic heterogeneity of *P. aeruginosa* in long-term biofilms in the lungs of patients with cystic fibrosis accelerate the evolution of phage resistance. This poses a major challenge to the durability of phage therapy [4]. This dynamic relationship, in turn, facilitates the long-term coexistence and coevolution of phages and bacteria within biofilms at the ecological level. Biofilms provide phages with a relatively stable, bacteria-rich ecological niche. High bacterial cell density increases encounter rates between phages and their hosts. Although the EPS matrix acts as an initial physical barrier, partial enzymatic degradation by phages creates accessible channels for phage invasion. The matrix also limits excessive diffusion of phage particles, thereby maintaining high local infective pressure [6]. For bacteria, the biofilm microenvironment represents a hotspot for horizontal gene transfer. Phage-mediated transduction, including generalized transduction and specialized transduction by temperate phages, enables efficient genetic exchange within biofilms [5]. This process drives the generation of genetic diversity in natural bacterial populations. Meanwhile, phages can also evolve their infection strategies by capturing host genes encoding depolymerases [3]. Therefore, biofilms can be regarded as hubs for genetic information exchange. There, phages serve as both important selective pressures and vehicles for gene flow, collectively shaping the genomic landscape of microbial communities. Ultimately, this complex interactive network, featuring antagonism and synergy, profoundly affects the composition, stability, and function of the entire microbial community. An in-depth understanding of such ecological and evolutionary interactions is essential for the rational design of phage therapy.

In addition to interactions between virulent phages and bacteria within biofilms, temperate phages can integrate into the host bacterial genome and profoundly reshape host phenotypes, including the ability to form biofilms. This process is called lysogenic conversion. Integrated prophage genes may encode novel regulatory factors or structural proteins, thereby modulating biofilm formation. For example, the VqmA protein encoded by prophage VP882 binds 3,5-dimethylpyrazin-2-ol (DPO) produced by Vibrio cholerae. This interaction initiates the phage lytic cascade through an anti-inhibition mechanism and also indirectly regulates expression of the host biofilm-associated gene VqmR [61]. Phage-encoded auxiliary metabolic genes are involved in DNA repair, quorum sensing, and EPS biosynthesis. These genes enhance microbial environmental adaptability by mediating antioxidant stress responses and maintaining biofilm stability [62]. Moreover, expression of the prophage gene Dap1 in *P. aeruginosa* attenuates bacterial motility and facilitates biofilm formation by interfering with DipA, a c-di-GMP phosphodiesterase [63]. Collectively, prophages predominantly promote bacterial biofilm formation through their own functional genes. This phenomenon is analogous to viral interference: enhanced biofilm formation favors replication of prophage genomes and production of progeny phages.

Overall, phages and biofilms form a dynamic antagonistic and synergistic coevolutionary system driven by ecological adaptation and genetic interaction. Virulent and temperate phages jointly regulate biofilm development and microbial genetic diversity. Further exploration of the molecular regulatory mechanisms underlying these interactions will help address phage therapy resistance and advance the precise application of phage-based antibacterial strategies in clinical and environmental settings. It should be noted that temperate and lysogenic phages may carry and transfer virulence or antimicrobial-resistance genes through transduction, and therefore require careful safety evaluation, including genome sequence validation and precise definition of host range, before therapeutic use.

## 3. Common Mechanisms of Phage and Their Derivatives Against Biofilms

**Figure 1:**
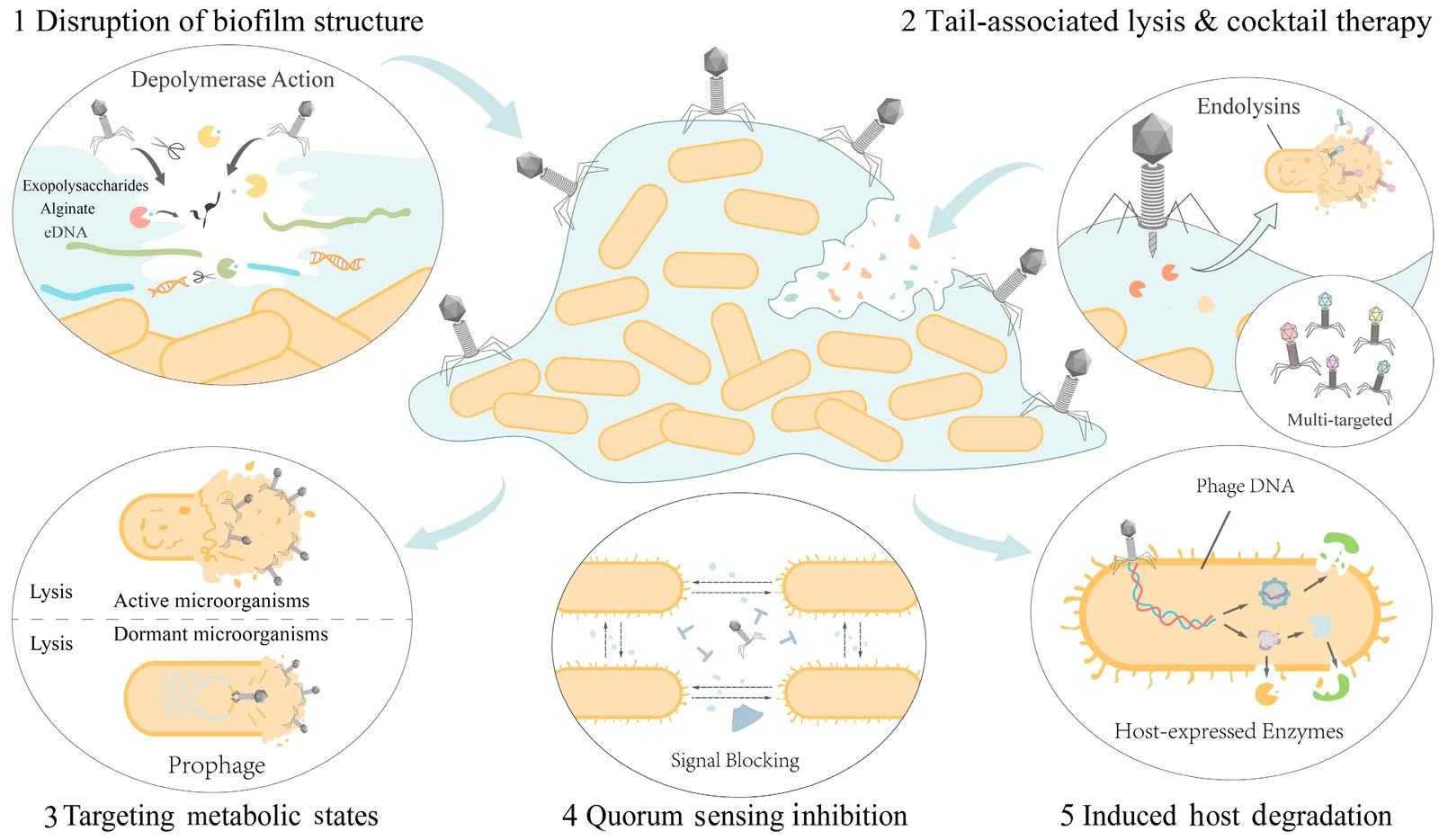
Common mechanisms of phage destruction and lysis of biofilms. (1) Biofilm degradation by phage depolymerases. These enzymes hydrolyze key biofilm matrix components (extracellular polysaccharides, alginate, and eDNA), destroying the protective barrier, increasing biofilm permeability, and enabling phage access to embedded bacteria. (2) Lysis by phage tail-associated enzymes and cocktail therapy. Tail fiber enzymes degrade biofilm EPS to facilitate phage diffusion, while endolysins lyse bacteria from within. Phage cocktails with multiple targets are used to overcome bacterial resistance and enhance efficacy. (3) Targeting bacteria in different metabolic states. Lytic phages primarily target active bacteria, while prophages in dormant persister cells can be activated by environmental stress to initiate new lytic cycles, eliminating both active and persistent populations. (4) Quorum sensing inhibition. Phages interfere with bacterial intercellular communication by secreting inhibitors or via depolymerase degradation products, suppressing biofilm formation and maintenance. (5) Induced host self-degradation. Phages induce host bacteria to express endogenous EPS-degrading enzymes.

Currently, five core mechanisms explain phage-mediated biofilm disruption and bacterial lysis (Figure 1). First, phage-encoded depolymerases degrade the biofilm matrix. These enzymes hydrolyze biofilm EPS and bacterial surface polysaccharides. This degradation removes the protective barrier around embedded bacteria, enabling phage infection and biofilm breakdown [64,65,66,67,68,69]. Depolymerases are key anti-biofilm effectors. They disrupt biofilm structure, improve matrix permeability, and inhibit biofilm formation by interfering with bacterial quorum sensing. Exogenous expression and genetic engineering of depolymerases support the development of novel anti-biofilm enzyme therapies [38,70,71,72,73,74]. Second, phage tail-associated enzymes mediate bacterial lysis. Phage tail fiber enzymes degrade biofilm EPS and bacterial cell walls [75,76,77,78]. Tail structural variation and fiber-EPS binding facilitate phage diffusion and local matrix degradation. Subsequent expression of endolysins and holins triggers intracellular bacterial lysis and cascading infection, ultimately causing biofilm collapse [3,5,79,80]. Phage cocktails with diverse characteristics can overcome bacterial resistance and achieve superior multi-target anti-biofilm efficacy [38,81,82]. Third, phages selectively lyse non-dormant biofilm bacteria. Dormant persister cells resist phage lysis, but phages can reinfect and lyse these cells once they resume growth [83,84,85]. Metabolic heterogeneity in biofilms enables bacterial dormancy and immune evasion. In addition, stress-induced prophage lytic cycles initiate in situ biofilm disruption [4,82]. Fourth, phages inhibit bacterial quorum sensing. Phage-secreted inhibitors such as lactonases block bacterial intercellular communication and suppress bacterial growth [86,87,88,89]. Degradation products generated by phage depolymerases and phage-encoded regulatory factors disrupt quorum sensing pathways. Temperate phages also inhibit biofilm formation by interfering with host regulatory networks [72]. Fifth, phage infection induces host bacteria to express endogenous EPS-degrading enzymes [90,91].

Classic studies have systematically summarized the conventional anti-biofilm mechanisms of phages and their derivatives. These mechanisms include matrix degradation, selective bacterial lysis, and quorum sensing inhibition. They remain dominant research directions in current investigations. Nevertheless, recent advances have revealed previously unrecognized functional patterns. Therefore, exploring novel anti-biofilm mechanisms of phage-based agents is essential to complement existing theoretical systems.

## 4. Novel Mechanisms of Phages and Their Derivatives Against Biofilms

### 4.1. Novel Quorum Sensing Interference Mechanism Mediated by Phages

The bacterial quorum sensing system serves as a core communication network that regulates biofilm formation and maturation. Bacteria can enhance biofilm formation via their own quorum sensing systems, thereby inhibiting phage adsorption [92]. Recent studies have demonstrated that phages actively interfere with host quorum sensing systems through multiple sophisticated mechanisms [72,93]. This interference profoundly modulates biofilm dynamics and provides a new perspective for deciphering phage–biofilm interactions and developing innovative anti-infection strategies. *V. anguillarum* uses N-acyl homoserine lactone (AHL)/quorum sensing-dependent anti-phage strategies in response to cell density. At low cell density, the bacterium highly expresses OmpK, which increases susceptibility to phage infection. At the same time, it enhances biofilm formation to mask phage receptors. At high cell density, it downregulates OmpK expression and thereby acquires phage resistance [94,95].

**Figure 2:**
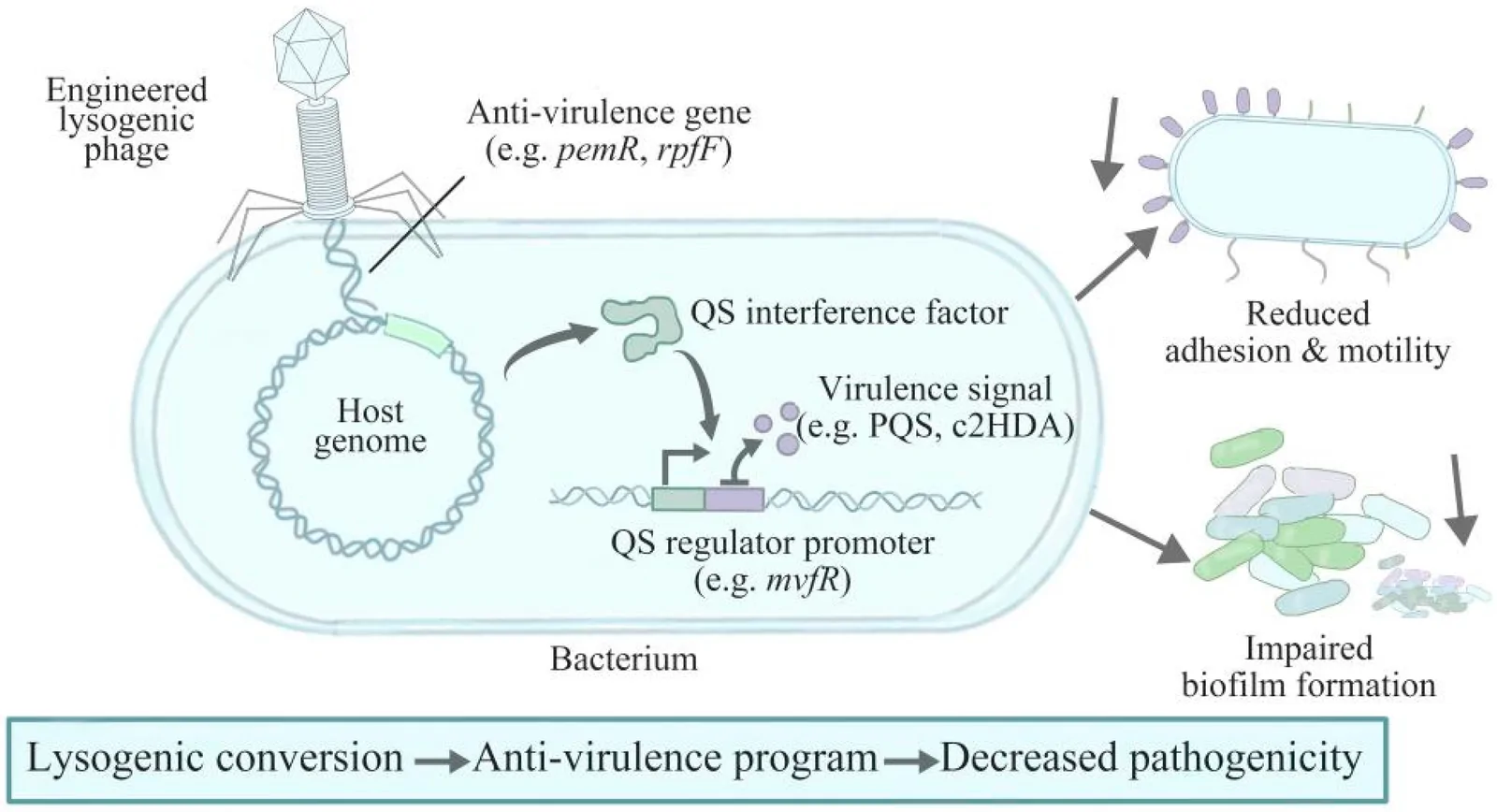
Prophage-mediated bacterial quorum sensing remodels the phenotypes. Engineered lysogenic phages act as gene delivery vectors, introducing anti-virulence genes to interfere with bacterial quorum sensing. This suppresses virulence factor production, motility, and biofilm formation, attenuating pathogenicity without direct bacterial lysis.

The mechanism by which phages interfere with bacterial quorum sensing systems that warrants the most research attention is lysogenic conversion. It enables long-term remodeling of bacterial phenotypes by integrating genes that encode quorum sensing-interfering factors into the host genome (Figure 2). Studies have confirmed that the transcription regulator PemR is encoded by phage PAYQ66. PemR directly binds to and represses the promoter of mvfR, a key quorum sensing regulatory gene in *P. aeruginosa* [72]. This repression reprograms bacterial metabolic flux and reduces biosynthesis of the virulence factor Pseudomonas quinolone signal (PQS). It also upregulates the global regulatory gene *rsmA* [72]. Consequently, it broadly inhibits multiple virulence phenotypes, including pyocyanin production, rhamnolipid synthesis, bacterial motility, and biofilm formation. After modification, the engineered temperate phage P22 targeting *Salmonella* tends to establish lysogeny. It carries the *rpfF* gene, which encodes the enzyme required for synthesis of the quorum sensing signal molecule cis-2-hexadecenoic acid (c2HDA) [96]. Lysogenic *Salmonella* continuously produces c2HDA. This molecule strongly inhibits the expression of virulence genes in the bacterium itself. It also suppresses the invasiveness of surrounding homologous bacteria via secreted c2HDA. As a result, c2HDA markedly reduces intestinal colonization and inflammatory responses in animal models [96]. These findings indicate that lysogenic phages can serve as efficient gene delivery vectors to precisely deliver virulence-inhibitory programs into pathogenic bacteria. Quorum sensing interference mediated by lysogenic phages has important therapeutic value. It supports a novel therapeutic strategy that is independent of bacterial lysis. Unlike bactericidal antibiotics or lytic phages, this strategy focuses on attenuating bacterial virulence rather than directly killing pathogens. Theoretically, it helps the host immune system and antimicrobial agents eliminate pathogenic bacteria efficiently. Furthermore, in complex multispecies infectious microenvironments, interference with the quorum sensing systems of key pathogens can disrupt interspecific communication networks. It can also regulate the organization and function of the entire microbial community. Thus, such interference provides tools for intervention strategies based on ecological principles. In the future, mining, modifying, and rationally applying phage-encoded quorum sensing-interfering factors will become an important direction for developing next-generation intelligent antibacterial therapies.

### 4.2. Promoting Penetration and Synergistic Efficacy of Antimicrobial Agents

The dense EPS matrix of biofilms acts as a critical physical barrier that hinders antibiotic penetration and ultimately causes therapeutic failure [97]. Phages can disrupt this barrier through direct lytic activity or through phage-encoded matrix-degrading enzymes. This disruption creates accessible channels for antibiotics and other antimicrobial agents to infiltrate the biofilm. The resulting “unlocking” effect markedly reduces matrix density and viscosity. Consequently, antibiotics that inherently penetrate biofilms poorly, such as aminoglycosides and macrolides, can diffuse more deeply into biofilm layers and reach a greater number of protected bacterial cells [98,99]. Aminoglycosides rely on active transport and aerobic metabolism for their bactericidal efficacy. Studies have demonstrated that this physical disruption underlies Phage–antibiotic synergy (PAS). This disruption can lower the minimum inhibitory concentration of antibiotics against biofilms by severalfold or even tenfold or more [100]. On the basis of these mechanisms, PAS has emerged as an actively investigated but still incompletely standardized strategy for combating refractory biofilm-associated infections. Many in vitro and animal model studies have validated its potent synergistic effects. In chronic lung infections caused by *P. aeruginosa* and in diabetic foot ulcer infections, phages combined with antibiotics such as tobramycin, ciprofloxacin, and meropenem produce more thorough biofilm elimination and lower bacterial burden than monotherapy [99]. Similarly, combined phage–antibiotic treatment shows synergistic or additive effects against biofilm-related infections caused by carbapenem-resistant *K. pneumoniae* and methicillin-resistant *S. aureus*. This combination may also alleviate the evolutionary pressure for the development of bacterial drug resistance [100,101]. PAS enhances bactericidal efficiency. It also delays resistance emergence and reduces the risk of resistance to either agent, but these effects depend on the bacterial strain and treatment regimen [102]. In favorable settings, this benefit arises because bacteria must simultaneously adapt to two distinct modes of action. Importantly, PAS does not universally prevent resistance. Its magnitude and even direction depend strongly on the bacterial strain, phage, antibiotic, dose, sequence and timing of administration, and experimental model. Resistance to one or both agents can still emerge under suboptimal regimens. Investigators should therefore optimize PAS empirically for each infection rather than assume that it is broadly protective. Overall, PAS provides a promising, multi-mechanism framework for addressing chronic and recurrent biofilm-associated infections.

### 4.3. Prophage Induced Disintegration and Dispersion of Biofilm Structures

Phages can trigger biofilm structural dissociation and active bacterial dispersion by inducing physiological reprogramming of the bacteria themselves. This mechanism represents a sophisticated targeted regulatory strategy. It is closely associated with the lysogenic cycle of phages and the expression of specific functional genes. Phage infection does not merely destroy bacterial structures. Instead, it drives bacteria to express or activate dispersion-related signaling factors, degrade intercellular adhesive substances, and detach spontaneously from mature biofilms into a planktonic state. The core of this mechanism is the integration of prophages into the bacterial genome during lysogenization. This integration modulates endogenous bacterial signaling pathways and virulence programs. It does not rely on exogenous physical or chemical stimuli and can thereby cause internal collapse of biofilm integrity. Studies on recombinant lysogenic phages of *Salmonella* have revealed that this regulation relies heavily on the endogenous synthesis of quorum-sensing signaling molecules [96]. Engineered lysogenic phages can carry key synthetic genes that induce host bacteria to autonomously produce c2HDA. As a potent virulence-suppressing signal, c2HDA directly silences the expression of bacterial invasion-related genes. It also interferes with intercellular communication required for biofilm maintenance, impairs biofilm structural stability, inhibits bacterial adhesion and colonization, and ultimately facilitates biofilm dissociation [96]. Future therapeutic strategies should account for the regulation of bacterial dispersion. Rational tailoring of engineered lysogenic phages could induce biofilm dispersion while rendering dispersed bacteria susceptible to immune clearance or subsequent antimicrobial intervention. This approach may transform potential dissemination risks into therapeutic opportunities.

## 5. The Tripartite Interaction of Phage, Bacteria and Host in the Microenvironment

### 5.1. Synergy and Regulation of the Host Immune System

Phage therapy against biofilm infections depends not only on direct phage–bacteria interactions but also on the host immune system. The host immune system and phages have complex synergistic and regulatory interactions. This tripartite interplay among phages, bacteria, and the host immune system determines the final therapeutic outcome [4].

Two opposing immune effects warrant clear distinction. The host immune system recognizes and clears phage particles. This clearance poses critical challenges to phage pharmacodynamics and pharmacokinetics in vivo. As exogenous viral particles, phages can trigger host antiviral immune responses. Under repeated or long-term administration, the host may produce neutralizing antibodies. These antibodies accelerate phage clearance from the blood circulation and reduce phage bioavailability at biofilm infection sites [4,81]. Therefore, understanding such clearance and then modulating or evading it may improve clinical efficacy. Strategies include screening for low-immunogenicity phages, using engineered capsids, and optimizing administration routes and regimens. On the other hand, existing studies have confirmed that natural phages can directly regulate host innate and adaptive immune responses [103]. These findings provide fundamental theoretical support for the engineering concepts described below. We postulated a purely hypothetical, not-yet-tested design concept: genetically engineered phages could be modified into smart vectors. These vectors would integrate delivery functions and immunomodulatory activities. They might reconcile the conflict between phage-mediated bacterial killing and host immune responses. They might also enable precise targeted intervention against biofilm infections in preclinical models [104]. The core design principle of this hypothesis was to endow phages with dual functional attributes. Such phages would lyse pathogenic bacteria and degrade the biofilm matrix while simultaneously remodeling the local immune microenvironment in situ. This approach could achieve an integrated intervention effect of antimicrobial activity, biofilm disruption, and immune co-regulation. Reported delivery systems include exosome-mediated miRNA delivery that modulates macrophage inflammation. They also include mesenchymal stem cell-based carriers that release anti-inflammatory cytokines to ameliorate lesional microenvironments [105]. Drawing on these systems, we further hypothesized that gene-edited phages could be loaded with immunomodulatory molecules, including chemokines and anti-inflammatory cytokines. Such loading could simultaneously achieve bidirectional immune regulation. From a theoretical perspective, we expected these remodeled phage vectors to recruit peripheral immune cells to infiltrate biofilm lesions and strengthen host clearance capacity. We also expected them to alleviate excessive local inflammatory responses, mitigate immune-mediated tissue damage, prolong phage retention at the infection site, and improve in vivo safety. We reiterate that all of these engineered immunomodulatory phage concepts remain at the proposal stage. To date, no complete in vitro multispecies biofilm models, in vivo animal infection experiments, or clinical data exist to corroborate these predictions. The relevant efficacy and in vivo safety await rigorous experimental validation.

In summary, constructing immunomodulatory engineered phage vectors that target biofilms remains a theoretical, preclinical-stage concept. This approach has not yet been validated in vitro or in vivo. In principle, such vectors could achieve targeted bacterial killing, biofilm matrix degradation, and local immune remodeling. If validated, this approach could help alleviate the antagonism between phage administration and host immunity. It could also provide a novel intervention strategy for MDR bacterial biofilm infections.

### 5.2. Niche Competition in Multispecies Biofilms

In complex multispecies biofilms, phage–bacteria interactions are embedded in a dynamic microbial ecological network. Phages can target dominant bacterial strains and reshape the species balance and spatial structure of the community. This reshaping affects biofilm stability and susceptibility to eradication [106]. In multispecies biofilms, the matrix secretion and cellular arrangement of nonhost bacteria can physically shield susceptible bacteria from phage invasion. In a dual-species biofilm model of *E. coli* and *V. cholerae*, susceptible *E. coli* cells embedded in the compact basal structure formed by *V. cholerae* acquire substantial protection against T7 phage. This protection occurs even though the *E. coli* cells do not produce a mature protective matrix [106]. This cross-protection results from the *V. cholerae*-modulated spatial architecture of the biofilm. This architecture restricts contact between phages and target host cells. Furthermore, phage therapy faces additional challenges in multispecies niches. The antibacterial efficacy of phages targeting *P. aeruginosa* declines rapidly after repeated exposure to *P. aeruginosa* biofilms [107]. Bacteria develop persistence through multiple mechanisms, including receptor mutation and the formation of high-alginate-tolerant phenotypes. These heterogeneous variations within biofilm populations weaken the sustained efficacy of phage therapy [107]. Mixed-species biofilms of *P. aeruginosa* and *Candida albicans* commonly occur in the lungs of patients with cystic fibrosis. Studies have indicated that the *P. aeruginosa* phages Pf4 and Pf1 can inhibit biofilm formation and planktonic growth of *C. albicans*, possibly through iron competition [108]. This finding illustrates a cross-species interaction in which phages indirectly regulate one microorganism by affecting another. It further confirms the complexity of interactions in multispecies biofilms.

In multispecies biofilms, phage intervention alters species balance. It may also inadvertently select for more tolerant or resistant bacterial subpopulations, such as persister cells [109]. Understanding these complex community interactions is a prerequisite for designing rational phage therapy. Rational phage therapy requires a shift from simple pathogen targeting to regulation of the overall microbial ecosystem. First, precise diagnosis of the microbial composition at the infection site is necessary to identify key bacterial species and their interactions. Second, researchers should fully consider the complexity of the microbial community when designing phage cocktail therapy. For instance, phages targeting different key bacterial species can be combined. Alternatively, researchers can select phages with a broad host range or with genes encoding multiple degrading enzymes. These phages can synergistically disrupt the EPS matrix produced by various bacterial species [110]. Finally, PAS is an important strategy for addressing the challenges of multispecies interactions. PAS can eliminate biofilms more effectively than monotherapy. For example, in biofilms formed by drug-resistant *S. aureus* or *P. aeruginosa* [111,112], combined therapy has shown stronger bactericidal and antibiofilm effects than monotherapy in both in vitro and in vivo models. In a strain- and regimen-dependent manner, it can also delay the emergence of bacterial resistance [111,112]. Such combination strategies help reshape the microbial community more thoroughly. They also help prevent the rebound of a single species. In conclusion, phages should be evaluated within the ecological context of multispecies biofilms. Therapeutic strategies should evolve from simple bactericidal action to ecological regulation. This shift is key to improving the clinical efficacy and durability of phage therapy.

### 5.3. Influence of Physicochemical Microenvironment Inside Biofilms on Phage Lysis of Host

**Figure 3:**
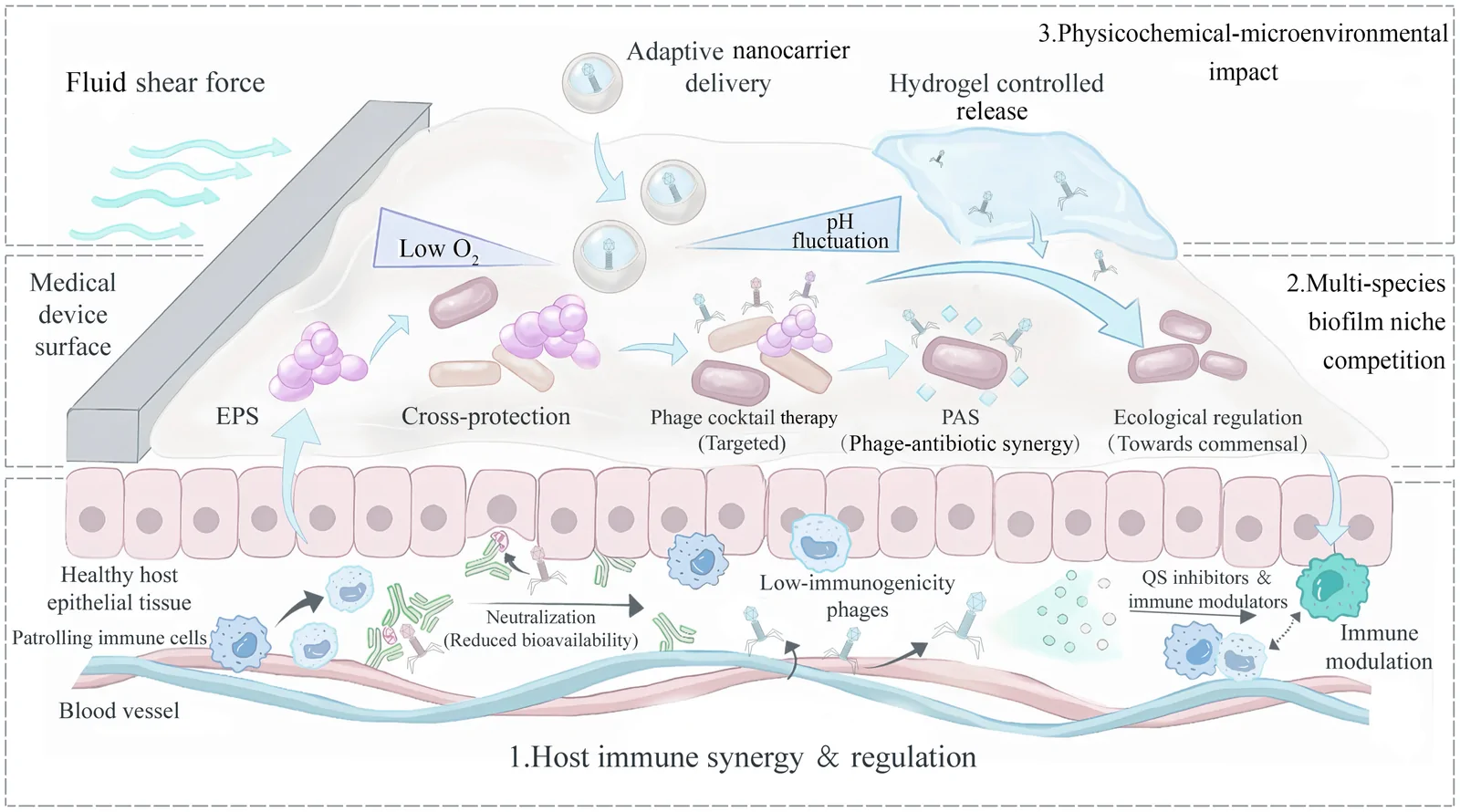
Physicochemical microenvironment regulating phage efficacy in biofilm. The tripartite interactions among phages, bacteria and the host microenvironment determine therapeutic outcomes and are organized into three layers. (1) Host immune synergy and regulation: within the healthy epithelial tissue and blood-vessel niche, phages are neutralized by circulating immune cells, which reduces phage availability; local innate immunity, however, can act synergistically with phages. (2) Multi-species biofilm niche competition: interspecies competition, cross-protection and ecological regulation further constrain phage activity. (3) Physicochemical microenvironment impact: low oxygen, pH fluctuation, fluid shear force on medical-device surfaces, and EPS-mediated diffusion barriers limit phage penetration, stability and replication. Strategies to overcome these barriers—adaptive nanocarrier delivery, hydrogel-controlled release, targeted PAS, and quorum-sensing/immune-modulating smart vectors—are shown as interventions that enhance biofilm eradication.

Figure 3 shows that the unique physicochemical microenvironment within biofilms is a key determinant of phage intervention efficacy. The heterogeneity of this microenvironment directly affects phage adsorption, penetration, and replication, thereby influencing final bactericidal efficacy.

The pulmonary biofilm microenvironment in patients with cystic fibrosis is complex and harsh. The highly viscous mucus, low oxygen tension, and metabolically dormant subpopulations of bacteria within this microenvironment pose major obstacles to phage therapy [4]. This dormancy severely restricts phage adsorption and intracellular replication. This restriction likely explains why phage therapy in clinical studies produces only transient reductions in bacterial burden and fails to eradicate infections [109]. In addition, pH fluctuations within biofilms may affect phage stability. Although individual phages can remain stable over a broad pH range, this in vitro stability still requires careful evaluation in the dynamic and complex in vivo microenvironment [22].

The hydrodynamic environment is another critical physical factor. The flow of body fluids or air continuously generates shear force on biofilms that form on medical device surfaces or in body cavities. On the one hand, shear force shapes biofilm structure. On the other hand, it determines whether phage particles can effectively reach and remain at the infection site. High shear forces may rapidly wash away phages and reduce their local effective concentration. Therefore, phage inhalation therapy for respiratory tract infections must consider how the fluid environment affects delivery efficiency. For example, studies using physiologically relevant airway models and respiratory simulators have evaluated the survival and delivery efficiency of different phages during aerosol inhalation [6]. These approaches highlight the importance of simulating physiologically relevant hydrodynamic environments.

The intrinsic surface properties of medical devices, including material composition, roughness, hydrophobicity, and surface charge, form the initial interface for biofilm formation. These properties indirectly regulate the efficacy of phage intervention. Rough or porous surfaces provide more anchoring sites for bacterial adhesion and the embedding of EPS. This property facilitates the growth of thicker, more complex biofilms, which may physically hinder deep phage penetration [2]. Therefore, future studies should simulate clinically relevant hydrodynamic and material-surface environments. They should explicitly account for and seek to overcome these physical factors when evaluating the anti-biofilm efficacy of phages.

The microenvironment has a decisive influence on phage therapy. Therefore, the development of adaptive local delivery systems has become a cutting-edge direction for optimizing this therapy [113]. An ideal delivery system protects phages from inactivation by adverse microenvironmental factors such as extreme pH and proteases. It also promotes phage retention and controlled release within biofilms [114]. Future therapeutic strategies must fully account for microenvironment-related factors. They should thoroughly analyze the unique microenvironment of targeted infection sites. Then, they should engineer phage preparations and corresponding delivery approaches accordingly [115]. This step is critical for overcoming current translational bottlenecks and achieving precise and effective treatment.

## 6. Rational Design Strategies for Potentiated Phage Therapy

### 6.1. Combined Therapy with Synergistic Enhancement via Multiple Mechanisms

The core of combination therapy is the integration of multiple antibacterial mechanisms. Through synergistic or additive effects, combination therapy overcomes the limitations of monotherapy and eradicates refractory biofilm infections more efficiently. Phage cocktail therapy is one of the most classic and widely adopted strategies. This approach combines phages that differ in host lysis spectra, lytic cycles, or encoded enzymes to achieve multiple goals: broadening the bactericidal spectrum to cover the heterogeneity of clinical isolates, preventing or delaying bacterial resistance to single phages, and disrupting biofilm structure more thoroughly through multi-target attacks. For example, in MDR *Salmonella*, a phage cocktail composed of three *Felixounavirus* strains (TSP_TW2 + TSP_SW1 + TSP_SJ5) not only delays the emergence of bacterial resistance for up to 24 h in vitro but also effectively eliminates biofilms [116]. Similarly, in MDR *P. aeruginosa*, a three-phage cocktail containing multiple functionally heterogeneous phages (EM + EC + 109) exhibits potent biofilm eradication activity when combined with antibiotics and prevents phage resistance [111]. Designing such combinations requires an in-depth understanding of the genomic characteristics and lytic kinetics of phages to optimize the synergistic effect of the combined formulation.

PAS represents another highly promising combined strategy. It is based on the principle that phages and antibiotics can produce a “1 + 1 > 2” therapeutic effect through distinct mechanisms. On the one hand, phages can lyse bacteria or disrupt the biofilm matrix, thereby markedly enhancing the killing efficacy of poorly permeable antibiotics against bacteria in deep biofilm layers [117,118]. For carbapenem-resistant *K. pneumoniae*, phage-based combination therapies have exhibited superior bactericidal effects compared with monotherapy both in vitro and in vivo [100]. On the other hand, subinhibitory concentrations of antibiotics can alter bacterial metabolism or the expression of cell surface receptors. This change promotes phage adsorption and replication, forming a positive feedback loop. Studies have confirmed that in MDR *E. coli*, pretreatment with phage ΦA3 for 7 h followed by amoxicillin-clavulanate treatment effectively eliminates bacteria in both planktonic and biofilm states and restores susceptibility to antibiotics [117]. For MDR *A. baumannii*, the combination of phage YC06 with antibiotics, including chloramphenicol, imipenem, or cefotaxime, produces strong synergistic bactericidal effects and can inhibit and eradicate mature biofilms [118]. For biofilms of MDR *E. faecium*, combining daptomycin and ampicillin with phage cocktails achieves satisfactory bactericidal outcomes without altering baseline antibiotic susceptibility or phage sensitivity [119]. In addition, for *P. aeruginosa*, combining phage φLCL12 with subinhibitory concentrations of imipenem significantly enhances biofilm inhibition and eradication in vitro and improves survival in a zebrafish infection model [120]. Nevertheless, the PAS effect is highly specific to bacterial strains, antibiotic types, and phage characteristics. Therefore, precise in vitro testing is required to guide the design of clinical regimens [121].

The combined application of phages and their derivatives with biofilm dispersants, antimicrobial peptides, and other agents constitutes a multi-target strategy against biofilms. Biofilm dispersants or specific enzyme preparations can destabilize EPS and physically loosen biofilm structures. This improves phage accessibility to bacteria embedded within the biofilm. Studies have shown that the phage depolymerase Dpo71 can inhibit biofilm formation and disrupt preformed biofilms. Dpo71 also significantly enhances the antibiofilm activity of colistin when used in combination [52]. Other studies support this combinatorial strategy. Lysostaphin (Lst) alone inhibits and disrupts *S. aureus* biofilms effectively. In contrast, Lst combined with DNase I shows markedly greater efficacy against *S. aureus* biofilms [122]. DNase I degrades extracellular DNA, interferes with biofilm formation, and disperses mature biofilms. Consequently, it facilitates Lst penetration. Looking ahead, combining phages and their derivatives with biofilm dispersants represents a promising but still largely preclinical strategy for developing novel antibiofilm agents.

In conclusion, successful combination therapy relies on an in-depth understanding of the complex interactions among phages, bacteria, and adjuvant agents within the biofilm microenvironment. It also requires rational design and personalized combination strategies based on this understanding.

### 6.2. Engineering Modification and Delivery System Design

As phage therapy moves from conceptual validation to clinical application, conventional natural phage preparations have revealed limitations in potency, stability, and controllability when targeting biofilms. Therefore, an in-depth understanding of phage–biofilm interactions is essential. Based on this understanding, researchers can optimize phage performance through genetic engineering. They can also innovate delivery systems to overcome in vivo microenvironment barriers. This combined approach will become a key frontier direction for improving therapeutic efficacy and expanding application scenarios.

Genetic engineering provides a powerful tool for customizing phages with enhanced antibiofilm capabilities. One core strategy is to strengthen the expression of phage-encoded depolymerases that degrade EPS. Using gene-editing technologies, researchers can upregulate endogenous depolymerase genes. Alternatively, they can heterologously introduce efficient depolymerase genes into phages that do not naturally encode these enzymes. These modifications can endow phages with stronger penetration and degradation abilities against biofilm matrices. The lungs of patients with CF present a complex mucus and biofilm environment. Screening or engineering phages that carry potent alginate lyase or DNase may significantly improve their diffusion and bactericidal efficacy within biofilms [4]. Another crucial engineering direction is to broaden the host range of phages. This direction addresses the high heterogeneity of clinical isolates and drug resistance that result from rapid bacterial evolution. Researchers can achieve this goal by modifying tail fiber proteins to recognize more diverse bacterial surface receptors. They can also construct phages that simultaneously express multiple receptor-binding proteins. In addition, removing non-essential proteins or modifying the capsid surface can reduce the risk of rapid clearance by the host immune system. This reduction is particularly important for treating chronic infections that require repeated administration. Beyond intact phage particles, purified phage-derived enzymes, especially endolysins and engineered depolymerases, exhibit unique advantages. These advantages include high specificity, a low propensity to induce bacterial resistance, lack of autonomous replication capacity, and favorable safety profiles.

Studies have demonstrated that phage combination therapy can effectively reduce the biofilm burden and inflammatory response of *P. aeruginosa* in ex vivo skin models and epidermal equivalent models under simulated physiological conditions [17]. These models provide an important preclinical validation platform for developing local delivery systems. Delivery systems aim to ensure that engineered or natural phages act continuously on biofilm lesions at an effective concentration and for an appropriate duration. Nanomaterials can encapsulate, capture, or adhere to specific phages. These actions enhance targeted delivery and therapeutic efficacy [104,123]. Functionalizing phages with nanomaterials to improve bacterial targeting can facilitate biofilm penetration and enhance antimicrobial potential [57]. Moreover, researchers have recently attached quantum dots to phages, creating a nanosystem that achieves precise bacterial targeting while maintaining phage infectivity. This approach enhances antibacterial activity and blocks bacterial replication. It achieves robust elimination of biofilm bacteria (over 99%) [124]. Furthermore, local sustained-release delivery systems show great potential for specific scenarios. These include chronic wound infections, implant-associated infections, and pulmonary infections in patients with CF. Hydrogels based on alginate, hyaluronic acid, chitosan, and other materials serve as ideal phage carriers because of their excellent biocompatibility and tunable physicochemical properties. They can immobilize phages at the infection site, maintain a moist healing microenvironment, and achieve controlled phage release to prolong the duration of action [15]. For orthopedic implant infections, incorporating phages or phage lysins into bone cement, hydroxyapatite coatings, or antibiotic-eluting scaffolds can form a localized antibacterial barrier on the implant surface. This barrier can prevent or treat early biofilm colonization [15]. For respiratory tract infections, aerosol inhalation of phage preparations is an effective strategy for directly targeting pulmonary biofilms. Optimizing aerosol particle size and stabilizing formulations to resist shear stress while preserving phage activity are currently major focuses of pharmaceutical formulation research [6].

**Figure 4:**
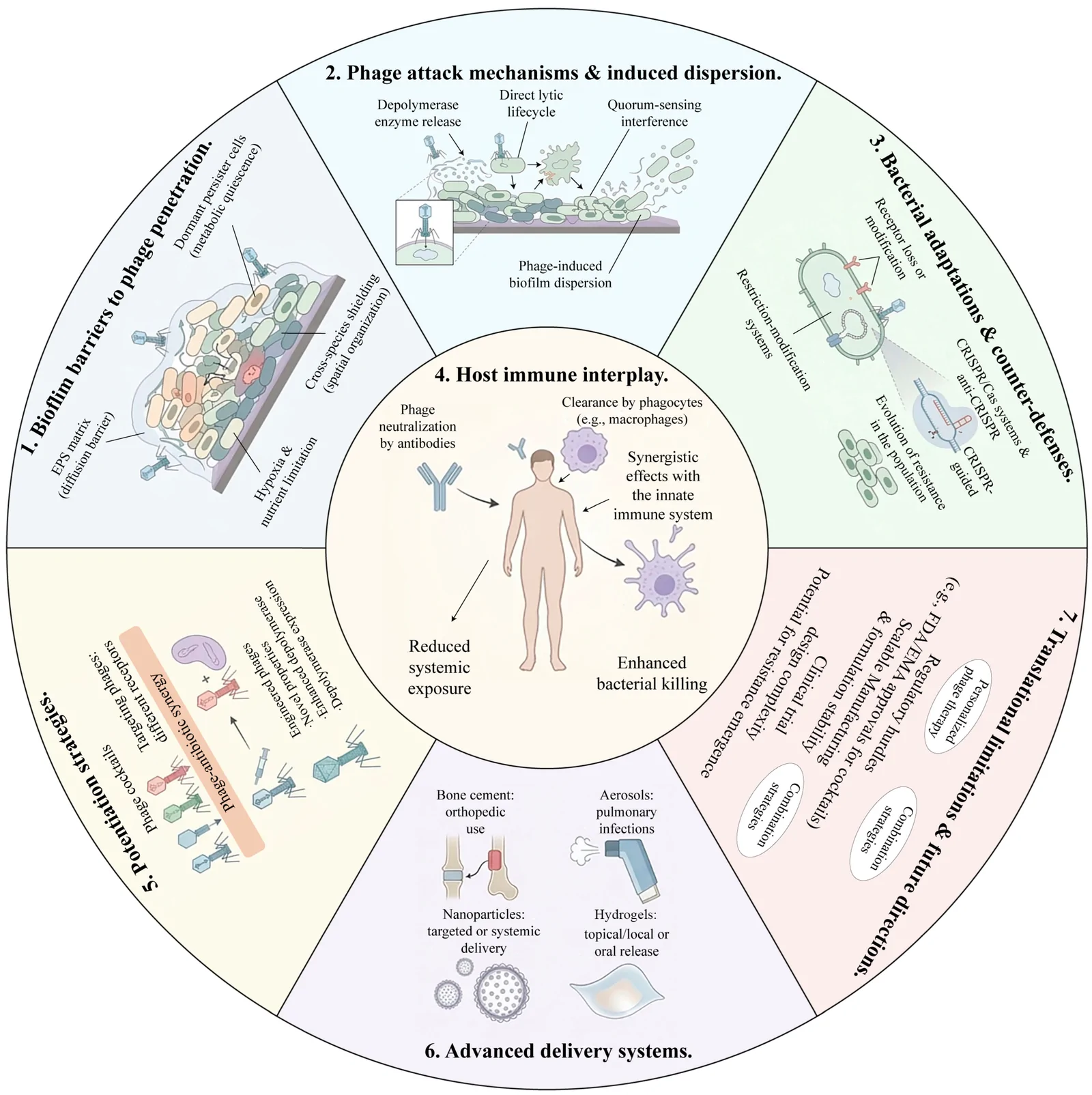
Proposed integrative schematic of phage—biofilm—host interactions and translational applications. The overview is organized as a mechanistic chain: (1) biofilm barriers to phage penetration, including the EPS matrix, dormant persister cells, hypoxia/nutrient limitation and cross-species shielding; (2) phage attack and dispersion, mediated by depolymerases, the lytic lifecycle, quorum-sensing interference and phage-induced dispersion; (3) bacterial counter-defenses, including receptor modification, CRISPR/Cas—anti-CRISPR, restriction-modification systems and resistance evolution; (4) host immune interplay, in which antibody neutralization and phagocytic clearance reduce systemic exposure while innate immunity synergizes with phage killing; (5) potentiation strategies, including receptor-targeted cocktails, phage—antibiotic synergy and engineered phages with enhanced depolymerase expression; (6) advanced delivery systems, comprising hydrogels, nanoparticles, aerosols and bone cement; and (7) translational limitations and future directions, covering personalized therapy, combination regimens, regulatory hurdles, manufacturing scalability, clinical-trial design and resistance emergence.

Figure 4 summarizes the phage–biofilm–host interaction as a closed loop. This loop spans biofilm barriers, phage attack mechanisms, bacterial counter-defenses, host immune interplay, potentiation strategies, and delivery systems. To achieve successful clinical translation, investigators must consider these components jointly.

## 7. Conclusions, Important Gaps, and Future Directions

Biofilm-associated bacterial infections are notoriously difficult to cure with conventional antibiotic regimens. The principal causes are the physical barrier formed by the EPS matrix, the metabolic heterogeneity within the biofilm, and the complex and diverse microstructure of the microenvironment. Phage therapy is a highly promising alternative antimicrobial approach. It can penetrate the biofilm barrier and specifically target MDR pathogens. Thus, it holds enormous potential for clinical translation in managing chronic and recurrent infections. Previous reviews have focused solely on the bactericidal and biofilm-eradicating efficacy of phages. In contrast, we systematically distinguished two types of dynamic interactions between phages and biofilms: antagonistic and synergistic. These interactions jointly determine the ultimate therapeutic outcome and the ecological consequences after phage intervention. Clarifying these differences is critical. This bidirectional regulatory relationship explains the dual role of phages in eliminating biofilms and reshaping the microbiome. It also illuminates the real-world discrepancy between ideal in vitro antibacterial effects and the instability of clinical efficacy.

Phage-mediated antagonism is a core mechanism underlying biofilm elimination and therapeutic efficacy. A large body of mechanistic studies have corroborated this role. The dense EPS matrix, the oxygen- and nutrient-depleted microenvironment, and the subpopulation of dormant persister cells jointly form a multilayered defense barrier. This barrier enables biofilm bacteria to evade antibiotic killing and host immune clearance. Lytic phages have evolved various strategies to breach these barriers. However, excessive attention to such beneficial effects risks overlooking the ecological adaptation between phages and bacteria. This adaptation profoundly shapes the long-term evolutionary trajectory of microbial communities.

At the therapeutic level, phages and biofilm-associated bacteria show antagonism. Conversely, they exhibit a synergistic and co-evolutionary relationship. This relationship has long been underestimated, but it directly dictates treatment failure and infection recurrence. The biofilm microenvironment provides phages with a high concentration of susceptible hosts and a stable niche. It prevents excessive phage dissemination and inactivation. It also sustains persistent local infectious pressure. From an evolutionary perspective, this niche adaptation promotes long-term coexistence between phages and bacteria. It drives bacteria to evolve multiple defense mechanisms, such as receptor mutations and anti-CRISPR systems. Phages, in turn, continuously expand their host range and optimize infection strategies. Most importantly, temperate phage-mediated lysogenic conversion can enhance bacterial biofilm fitness through horizontal gene transfer. Prophages integrated into the host genome modulate bacterial transcriptional networks. They alter motility, stress tolerance, and the efficiency of EPS synthesis. These changes promote biofilm formation and enhance structural stability. Such synergistic regulation may remodel the bacterial community after phages selectively eliminate susceptible bacteria. This remodeling can readily enrich persistent drug-resistant strains and opportunistic pathogens. Therefore, the coexistence of antagonistic and synergistic effects makes phage therapy a double-edged sword. Its use requires a balance between antibacterial potency and ecological safety.

The complex clinical infection microenvironment further amplifies the uncertainty of the phage–biofilm interaction network. This complexity is a major reason why laboratory findings are difficult to translate into clinical practice. Most in vitro experiments use simplified, static, single-species biofilm models. These models cannot recapitulate the authentic features of clinical multi-species biofilms, dynamic host immune responses, or fluctuating physicochemical microenvironments. In multi-species biofilms, non-host bacteria can cross-protect susceptible strains through physical shielding mediated by spatial structure. Interspecies niche competition can also alter phage activity, thereby compromising therapeutic stability. Host immune cells can cooperate with phages to eliminate pathogenic bacteria. However, neutralizing antibodies produced by the host can accelerate in vivo phage clearance and weaken long-term antibacterial effects. Microenvironmental stresses such as hypoxia, pH fluctuations, and fluid shear forces further inhibit phage replication. They disrupt virion integrity and impede the deep penetration of phages. Consequently, these stresses prevent complete eradication of the microbial community. Currently, our understanding of these multidimensional interaction mechanisms remains fragmented. This fragmentation makes it difficult to comprehensively assess the long-term stability and ecological risks of phage therapy.

The translational value of the available evidence is uneven. Most mechanistic findings come from in vitro biofilm models, which are often static and single-species. A smaller but growing body of evidence derives from ex vivo tissue and animal models, predominantly murine models, insect models, and small-animal implant systems. Robust controlled evidence in humans remains scarce. Most human evidence comes from case series, compassionate-use programs, and a few early-phase trials. Therefore, clinicians and researchers should interpret claims regarding efficacy, resistance suppression, and safety at the appropriate evidence level. They should not directly extrapolate observations valid only under defined in vitro conditions to patients.

Phage therapy must overcome several bottlenecks before it enters routine clinical use. It remains difficult to standardize and manufacture well-defined phage preparations with consistent titers and purity. In addition, phage pharmacokinetics and tissue penetration into deep biofilm sites remain incompletely understood. Repeated dosing can elicit neutralizing antibodies. Phage-resistant mutants and tolerant persister subpopulations can also emerge. Clinical biofilms differ markedly between and within patients. Consensus on dosing, treatment sequence, and route of administration is lacking. Regulatory and quality-assurance frameworks also remain unsettled. These caveats mean that even encouraging preclinical results may not translate reliably into routine clinical practice.

To address the aforementioned bottlenecks and achieve robust clinical translation, future research should move beyond the constraints of traditional research paradigms. It should develop targeted optimization strategies based on the mechanisms of bidirectional phage–bacterium interactions. In the long term, researchers should establish unified industry standards and conduct rigorous multicenter clinical trials. These trials should systematically validate the safety, efficacy, and long-term ecological impacts of optimized phage-based approaches. These efforts will promote the standardized and sustainable clinical implementation of next-generation anti-biofilm phage therapy.

## Figures and Tables

**Table 1:** Special studies on the efficacy of phage and phage-based preparations on bacterial biofilms in recent years.

Formulation Type	Formulation	Target Strain	Core Function and Effect	Reference
Phage	Kpn_PImp2, Kpn_PImp3	Carbapenem-resistant *K. pneumoniae*	Both phages exhibit potent capacity to inhibit and disrupt biofilm formation. At a concentration of 10^8^ PFU/mL, Kpn_PImp2 and Kpn_PImp3 can inhibit or eliminate approximately 50% of biofilm.	[33]
	PA-319, PA-575, PA-711	*P. aeruginosa*	Exert strong lytic activity and markedly inhibit/eradicate mature biofilm, with superior efficacy to high-concentration antibiotics.	[3]
	AbpL, AbT1	Multidrug-resistant (MDR) *A. baumannii*	AbpL effectively disrupted established biofilm, while AbT1 efficiently inhibited biofilm formation.	[8,9]
	T4	*Escherichia coli*	Lyses *E. coli* biofilms and serves as a classical model for phage-mediated antibiofilm strategies.	[34]
	En5822	Drug-resistant *Enterobacter cloacae*	Possess high lytic activity and strong specificity, and can significantly degrade biofilm.	[35]
	PSKP16	K2-type hypervirulent *K. pneumoniae*	For biofilms formed within 24 h and 48 h, PSKP16 at 10^8^ PFU/well reduced in vitro biofilm biomass by 18–64.6% and 16.4–63.7% within 24 h, respectively.	[36]
	Phage cocktail (PSCs1/PSDs1/PSCs2/PSSr1/PSMc1)	*Salmonella* Typhimurium	The lysis rate of biofilms on gallstone or tooth surface models reached 63.6%.	[37]
	Macy, Sally	*Raoultella planticola*	Phage Macy disrupted preformed *R. planticola* biofilms, reducing biomass by 56–71% relative to untreated controls. Phage Sally exhibited stronger eradication efficacy, lowering biofilm biomass by 82–85%.	[38]
	UHKP	*K. pneumoniae*	In static biofilm tests, phage UHKP at MOI = 1 removed 98% and 96% biomass of 24 h and 48 h biofilms within 24 h, while its clearance efficiency against 72–96 h mature biofilms was capped at 87%.	[39]
	vB_KpnA_SCNJ1-Z, vB_KpnA_SCNJ1-C, vB_KpnA_SCNJ1-Y	Carbapenem-resistant *K. pneumoniae*	Relative to controls, phage vB_KpnA_SCNJ1-Z (MOI = 100) reduced biofilm biomass by 80%. At MOI = 0.1, vB_KpnS_SCNJ1-C and vB_KpnM_SCNJ1-Y cut biofilm loads by 90% and 89%, respectively.	[40]
	pTJK	*Mammalian cocci*, *Staphylococcus aureus*	It can infect various *M. cocci* and *S. aureus*. It significantly inhibits biofilm formation and eradicates preformed biofilms even of non-host strains, and exhibits synergistic effects with gentamicin and erythromycin.	[41]
Endolysin	lysSEP21	*Salmonella* + *E. coli*	It can penetrate the outer membrane of Gram-negative bacteria without modification, reducing biofilm biomass by 90% within 6 h.	[42]
	Lys572, AVPL, Lys19	*Streptococcus suis*	Lys572 eradicates mature *S. suis* biofilms. AVPL suppresses 48 h *S. suis* biofilm formation and dismantles mature biofilms. Lys19 functions as a broad-spectrum endolysin targeting biofilms of *Clostridium perfringens* and *S. suis*.	[11,43,44]
	Lys08	*Enterococcus faecalis*	Lys08 showed antibiotic-membrane activity against *E. faecalis* biofilms, and this activity could be enhanced by Mn^2+^.	[45]
	CF-301	*S. aureus*	CF-301 clears mono- and mixed staphylococcal biofilms on medical substrates. It erases catheter biofilms in 1 h and detached bacteria in 6 h, and targets synovial fluid-derived *S. aureus* biofilm variants.	[46]
Depolymerase	PRA33gp45	*K. pneumoniae*	PRA33gp45 is a novel and highly specific depolymerase that enhances innate immune clearance by targeting its protective capsule, reducing *K. pneumoniae* toxicity, reducing biofilm persistence.	[47]
	Dep42	*K. pneumoniae*	It can inhibit the formation and degradation of biofilm formed by *K. pneumoniae*.	[48]
	Dep193	Multiple *Vibrio* species	Dep193 represents a novel clade depolymerase lacking canonical functional domains, with broad polysaccharide degradation and strong antibiofilm efficacy for aquaculture use.	[49]
	Dpo7	*S. aureus*	Dpo7 degrades matrix polysaccharides to loosen biofilm structure and facilitate the penetration of phage IPLA7.	[50]
	Dpo71	MDR *A. baumannii*	Dpo71 enhances serum bactericidal activity, effectively inhibits biofilm formation and eliminates established biofilms.	[51,52]
Engineered phage	Non-covalent phage-polymer nanoassemblies (PPNs)	*S. aureus*	PPNs retain the infectivity of phages and exhibit stronger biofilm penetration and bactericidal ability than free phages.	[53]
	φEf11/φFL1C (Δ36) (PnisA)	*E. faecalis*	Convert lysogenic phages into lytic ones to broaden host spectrum, reducing viable counts in biofilm by 10 to 100 folds.	[54]
Combination formulation	Phage vB_KpUKJ_2 + Ceftazidime	*K. quasipneumoniae*	It can rapidly and continuously reduce the viability of *K. quasipneumoniae* within 24 h in mature biofilms.	[16]
	Phage + cefiderocol	Gram-negative aerobic bacilli	Combination therapy exhibits superior antibiofilm efficacy compared with monotherapy using cefiderocol or phage alone, and can completely eradicate biofilms in certain cases.	[55]
	Phage + Silver nanoparticles	*S. aureus*	*Remove* preformed *S. aureus* biofilms from inert glass surfaces in a time-dependent manner.	[56]
	Phage + gold nanorods	*P. aeruginosa*	This system specifically targets *P. aeruginosa* biofilms. NIR irradiation of the phage-nanorod complex efficiently eradicates bacteria without notable epithelial cell injury.	[57]
	Endolysin LysPB32 + short peptides FK13/FK16	*Salmonella*	FK13 plus LysPB32 reduced biofilm OD_600_ from 0.6 to 0.16, while FK16 combined with LysPB32 lowered OD_600_ to 0.13.	[58]
	Endolysin LYSMP-HPP + Berberine hydrochloride	*S. suis*	The combined treatment significantly enhanced biofilm clearance and formation on porcine skin.	[59]
	Endolysin Lysin1902-HP-PC + Tea polyphenols	*E. coli*	The combination treatment efficiently eliminated biofilms on both polystyrene and stainless steel surfaces.	[60]

## Data Availability

No new data were created or analyzed in this study. Data sharing is not applicable to this article.
